# Use of Quorum Sensing Inhibition Strategies to Control Microfouling

**DOI:** 10.3390/md19020074

**Published:** 2021-01-30

**Authors:** Andrea Muras, Ana Parga, Celia Mayer, Ana Otero

**Affiliations:** 1 Departamento de Microbioloxía e Parasitoloxía, Facultade de Bioloxía-CIBUS, Universidade de Santiago de Compostela, 15782 Santiago de Compostela, Spain; andrea.muras@usc.es (A.M.); ana.parga.martinez@usc.es (A.P.); Celia.Mayermayer@nottingham.ac.uk (C.M.); 2 National Biofilms Innovations Centre, Biodiscovery Institute and School of Life Sciences, University of Nottingham, Nottingham NG7 2RD, UK

**Keywords:** microfouling, biofilm, marine bacteria, quorum sensing, quorum quenching, lactonase

## Abstract

Interfering with the quorum sensing bacterial communication systems has been proposed as a promising strategy to control bacterial biofilm formation, a key process in biofouling development. Appropriate in vitro biofilm-forming bacteria models are needed to establish screening methods for innovative anti-biofilm and anti-microfouling compounds. Four marine strains, two *Pseudoalteromonas* spp. and two *Vibrio* spp., were selected and studied with regard to their biofilm-forming capacity and sensitivity to quorum sensing (QS) inhibitors. Biofilm experiments were performed using two biofilm cultivation and quantification methods: the xCELLigence^®^ system, which allows online monitoring of biofilm formation, and the active attachment model, which allows refreshment of the culture medium to obtain a strong biofilm that can be quantified with standard staining methods. Although all selected strains produced acyl-homoserine-lactone (AHL) QS signals, only the *P. flavipulchra* biofilm, measured with both quantification systems, was significantly reduced with the addition of the AHL-lactonase Aii20J without a significant effect on planktonic growth. Two-species biofilms containing *P. flavipulchra* were also affected by the addition of Aii20J, indicating an influence on the target bacterial strain as well as an indirect effect on the co-cultured bacterium. The use of xCELLigence^®^ is proposed as a time-saving method to quantify biofilm formation and search for eco-friendly anti-microfouling compounds based on quorum sensing inhibition (QSI) strategies. The results obtained from these two in vitro biofilm formation methods revealed important differences in the response of biosensor bacteria to culture medium and conditions, indicating that several strains should be used simultaneously for screening purposes and the cultivation conditions should be carefully optimized for each specific purpose.

## 1. Introduction

Biofilms are made of microbial sessile communities characterized by cells that are attached to a substratum or other surfaces, are embedded in a self-produced matrix of extracellular polymeric substances, and exhibit an altered phenotype compared to planktonic cells [1]. Besides their consequences for human health, bacterial biofilms are based on important processes such as biocorrosion and constitute the initial step in the biofouling process of submerged surfaces, serving as the substratum on which other micro- and macro-organisms settle and grow. Biofouling control is a global economic problem due to its negative impact not only on the shipping industry (biocorrosion, cleaning, transport delay, increased fuel consumption) but also on environmental pollution (increased carbon dioxide, carbon monoxide, and sulfur dioxide emissions) [2]. There is increasing concern about the toxicity of biocides used to control marine biofouling since the biocide tributyltin (TBT) and other herbicides such as irgarol and diuron were banned because of their harmful impact on the marine ecosystem [3]. Most current commercially available antifouling paints contain compounds that are toxic to marine life, hence we need to search for new, less aggressive, eco-friendly substances to control biofouling and marine biofilm formation. Within that search, compounds targeting the inhibition of bacterial biofilm without affecting bacterial growth are of special interest. 

Understanding the mechanisms that control the bacterial adhesion process is essential to prevent the attachment of microorganisms onto surfaces [4]. Biofilm formation, as well as many other important biological processes in bacterial populations, is often coordinated through a cell density-dependent gene regulation system known as quorum sensing (QS) [5]. The QS system can induce changes in the production of exopolysaccharides, lipids, nucleic acids, and proteins that are components of the biofilm matrix. Furthermore, this bacterial communication system is integrated with other environmental signals, such as temperature, pH, salinity, oxidative stress, and nutrient availability, to improve the bacterial adaptation to the ecosystem and optimize their probability of survival [6,7]. Different types of QS signaling molecules have been described, and in some cases, several QS systems are present in the same species, making these cell-to-cell communication mechanisms highly specific and accurate. Moreover, acyl-homoserine-lactones (AHLs), the principal QS signals produced by Gram-negative bacteria that dominate marine biofilms, have been reported to modulate the settlement of zoospores of the marine alga *Ulva intestinalis* [8] and the cyprid *Balanus improvisus* [9] and promote the formation of diatom-biofilm [10], and are therefore involved in the macrofouling processes. 

Over the last years, interfering with QS bacterial communication systems has been proposed as a promising mechanism to prevent the development of different types of bacterial biofilms [11,12,13,14,15]. Commonly, the term “quorum quenching” (QQ) refers to the enzymatic inactivation of QS processes [16], while the more general term “quorum sensing inhibition” (QSI) is preferred to describe the chemical disruption of the cell-to-cell communication system caused by QS inhibitory compounds [17]. Blocking the bacterial QS systems to avoid bacterial colonization is a widespread strategy already used in nature by different kinds of organisms [16,17,18,19,20]. It has been demonstrated that eukaryotic organisms such as the red alga *Delisea pulchra* have developed cheating molecules that mimic the QS to avoid bacterial fouling of the algal surface [20]. Also, many bacteria have developed QQ enzymes that degrade QS signals [21,22,23,24], and this is the most-studied QS interference strategy. Using QQ enzymes that can degrade the AHLs produced by Gram-negative bacteria has been suggested as a novel anti-biofouling strategy [25,26,27], and a few studies have evaluated the effect of QQ enzymes on the ecology of multispecies biofilms [14,28,29,30]. QQ strategies have been successfully applied to control biofouling in membrane bioreactors (MBRs) for wastewater treatment [11,15] and to reduce biocorrosion [28], as they are associated with deep changes in the microfouling community [28]. This effect could be observed even in in vitro oral biofilm models dominated by Gram-positive species [14]. Moreover, QQ enzymes have been proposed as an interesting alternative to antimicrobial compounds because the probability of inducing tolerance or resistance against these mechanisms is lower [31,32], since the strategies based on silencing QS systems do not directly interfere with bacterial growth, therefore their use constitutes an eco-friendly strategy. 

Since the fact that QS-related genes are required for the successful establishment of marine microbial communities is generally accepted [33], and increasing evidence is being accumulated regarding the role of QS in marine bacteria involved in biocorrosion [34], appropriate in vitro marine bacterial biofilm models are needed to screen innovative anti-biofilm and anti-microfouling compounds based on QSI. Most in vitro models are based on monospecific biofilms, and the choice of bacterial strains depends on the objective of the bioassay [35,36,37]. Species of the genus *Pseudoalteromonas*, *Cobetia*, or *Shewanella* have been used as models for marine biofilms [36,38,39,40,41,42], although little information is available on the role of QS in biofilm formation in these species. Moreover, it should be taken into account that the culture conditions can have a significant impact on QS-QQ systems, biofilm formation, and/or surface attachment [12,22,23]. Due to this complexity, new methods are needed to understand and characterize bacterial biofilms [43] and identify novel anti-biofilm compounds. 

Most common biofilm assays are based on spectrophotometric methods that quantify total biofilm formation in 96-well microtiter plates. Although these biofilm assays are simple and do not require specialized equipment [44], the formation of biofilm in the microtiter plates is weak and highly variable and the quantification technique is quite time-consuming. Recently, the real-time monitoring system xCELLigence^®^, based on the measurement of impedance with sensors located on the bottoms of the wells of special microtiter plates, has been successfully applied to measure biofilm formation in the human Gram-positive pathogens *Staphylococcus aureus, S. epidermidis*, and *Streptococcus mutans* [13,45,46]. While the traditional biofilm quantification methods, such as the staining assays using crystal violet, the MTT (3-(4,5-dimethylthiazol-2-yl)-2,5-diphenyltetrazolium bromide) tetrazolium reduction assay, or the colony forming unit (CFU) estimation are end-point techniques, the xCELLigence^®^ system allows the continuous analysis of bacterial cell adhesion based on single-frequency impedance spectroscopy. However, the system is not suitable for measuring biofilms formed in the liquid/air interphase and has demonstrated low sensitivity for strictly aerobic human pathogens such as *Acinetobacter baumannii* compared to other biofilm measurement systems [12]. The xCELLigence^®^ system allows simple, high-throughput screening for novel anti-biofilm compounds, but it is necessary to assess whether it is suitable for accurate measurement of marine biofilms, since impedance could be affected by the high salinity required for marine bacteria cultivation. 

In the present study, we compare the xCELLigence^®^ real-time measurement system with the attachment model [14], a modification of the Amsterdam Active Attachment biofilm model [47,48], in which glass coverslips (18 × 18 mm) are immersed vertically in the culture medium in 12-well cell-culture plates. The attachment model allows refreshment of the culture medium without disturbing the biofilm, provides a higher adhesion surface (up to 1.62 cm^2^), and allows direct observation of structural changes in the biofilm, addressing some disadvantages of the use of 96-well microtiter plates [12].

The aim of this study was to evaluate biofilm formation by different marine bacteria and their sensitivity to QS inhibitors using these two in vitro cultivation systems with different culture conditions, in order to select suitable biosensor strains and establish a screening methodology for the identification of new anti-microfouling compounds based on QQ and QSI strategies.

## 2. Results

### 2.1. Selection of Marine Bacterial Biofilm-Forming Strains and Validation of xCELLigence^®^ Screening Method

A literature search was carried out to identify the marine bacterial strains most commonly used as model organisms in marine microfouling studies and/or reported to produce biofilm and AHLs. The search yielded a list of seven marine species: *Cobetia marina* (formerly *Halomonas marina*), *Shewanella putrefaciens*, *Pseudoalteromonas flavipulchra, P. maricaloris, Vibrio anguillarum, V. aestuarianus*, and *V. tubiashii* [36,38,39,40,41,42,49,50,51]. Strains were also selected based on the ability to grow on Tryptic Soy Broth (TSB) medium (NaCl concentration 0.5%), since initial experiments demonstrated that Marine Broth (MB), which has high salinity (total salt 3.4%, NaCl 1.9%), interferes with the sensors of the xCELLigence^®^ equipment. 

**Figure 1:**
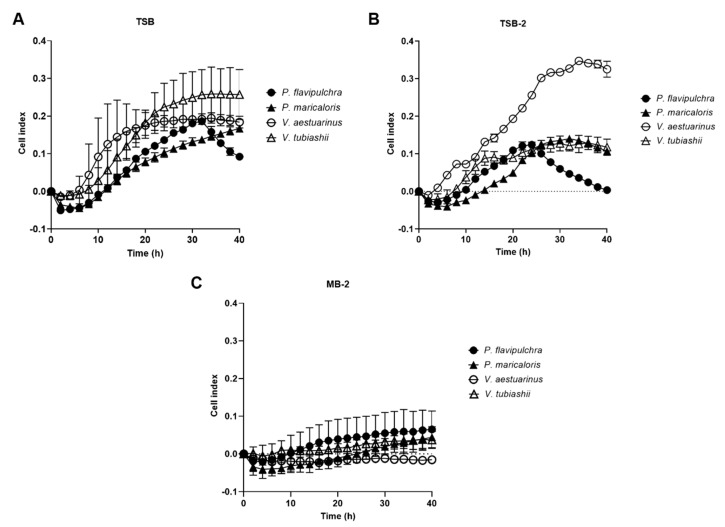
Biofilm formation of marine bacterial strains *Pseudoalteromonas flavipulchra, P. maricaloris, V. aestuarianus*, and *V. tubiashii* measured by xCELLigence^®^ system using (**A**) Tryptic Soy Broth (TSB) culture medium, (**B**) TSB with an NaCl concentration of 2% (TSB-2), and (**C**) Marine Broth (MB) adjusted to a total salinity of 2% (MB-2).

In order to select the best candidate marine strains to be used as biosensors for detecting anti-microfouling compounds, a preliminary biofilm quantification experiment was performed. Among the seven strains identified in the literature as models for biofouling studies that can grow in (Tryptic Soy Agar/Broth) TSA/TSB, *V. aestuarianus* and *V. tubiashii* had the best performance regarding biofilm formation in TSB as measured by xCELLigence^®^ (Figure 1A). A sharp decrease in cell index (CI) values, a unitless parameter correlated with an increase in the number of cells adhering to the bottoms of wells, of *P. flavipulchra* was observed at the end of the cultivation period (hours 34 to 36), indicating a biofilm detachment process. No significant biofilm formation could be detected by xCELLigence^®^ for *C. marina, S. putrefaciens*, and *V. anguillarum* in TSB (Supplementary Figure S1). Intermediate CI values were obtained for *P. maricaloris* and *P. flavipulchra* in TSB. Since high CI values are correlated with an increased number of cells adhering to the bottoms of wells in the xCELLigence^®^ system, *P. flavipulchra*, *P. maricaloris, V. aestuarianus*, and *V. tubiashii* were selected for further study. The use of TSB-2 (TSB supplemented with NaCl to a final concentration of 2%) caused a clear increase in the biofilm formed by *V. aestuarianus* as detected by the xCELLigence^®^ system, but lower CI values were observed for *P. flavipulchra* (Figure 1B), and the other two strains showed similar results. The biofilms cultivated with the MB-2 marine culture medium produced the lowest CI values in the xCELLigence^®^ system (Figure 1C). 

### 2.2. AHL Production by Marine Bacterial Biofilm-Forming Strains

In order to assess the production of AHLs by the four selected biofilm-forming marine strains, a qualitative analysis was performed first using the engineered *Chromobacterium violaceum* biosensors (Supplementary Figure S2). The production of violacein by the sensor *C. violaceum* VIR07 was activated by the supernatants of *V. aestuarianus* cultures, indicating the production of at least one long-chain AHL in high concentration (Supplementary Figure S2C). The induction of violacein production by VIR07 was not affected by the culture medium used for the cultivation of *V. aestuarianus* (MB, MB-2, TSB, and TSB-2). No activation of the long-chain AHL biosensor VIR07 was observed for any of the other bacteria in any of the culture media used (Supplementary Figure S2A,B,D). None of the bacterial strains were able to activate the short-chain biosensor CV026 in the plate assay (data not shown).

Since the *C. violaceum*-based biosensor plate assay presented some limitations, such as a high detection threshold and an inability to detect any 3-hydroxy-substituted AHLs [52], HPLC-MS analysis of the supernatants of the four strains cultured in MB was performed. The production of AHL was confirmed in all bacterial strains (Table 1). *P. flavipulchra* and *P. maricaloris* species presented similar AHL profiles, with the synthesis of six and five different AHLs, respectively. C4-HSL was the main AHL produced by both *Pseudoalteromonas* strains, reaching 374 and 347 ng/mL for *P. flavipulchra* and *P. maricaloris,* respectively. Both strains produced OC6, C8, and OC10-HSL. Slightly higher concentrations of OC6-HSL and OC10-HSL were found in *P. flavipulchra*. In accordance with the observed activation of the *C. violaceum* VIR07 biosensor strain, *V. aestuarianus* synthetized a broad variety of AHLs (11 different types) of different lengths, from C4-HSL to C16-HSL, with OC10-HSL as the main QS signal (2958 ng/mL). On the contrary, *V. tubiashii* only produced short-chain C4-HSL (503 ng/mL). 

### 2.3. Characterization of Quorum Quenching Activity of Marine Bacterial Biofilm-Forming Strains

**Figure 2:**
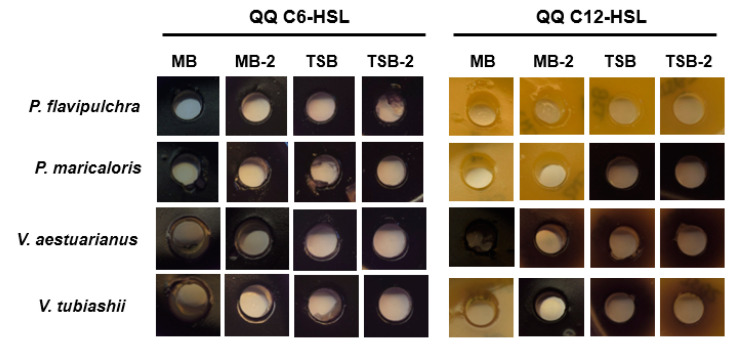
Quorum quenching activity of four selected biofilm-forming marine strains cultured in different media (MB, MB-2, TSB, and TSB-2) against short-chain signal C6-HSL and long-chain C12-HSL.

The capacity of the four biofilm-forming marine strains to interfere with short-chain (C6-HSL) and long-chain (C12-HSL) AHLs was tested using a *C. violaceum*-based bioassay. The ability to quench C12-HSL was observed in *P. flavipulchra* in the four different culture media used (Figure 2). However, *P. maricaloris* was able to interfere with C12-HSL only when it was cultured in MB and MB-2, losing this QQ activity in TSB and TSB-2 culture media. *V. tubiashii* was also able to quench C12-HSL, but only in MB. None of the four selected bacteria were able to degrade C6-HSL in the tested conditions.

### 2.4. Effect of QS Inhibitors and QQ Enzymes on Biofilm Formation Measured using xCELLigence^®^

**Figure 3:**
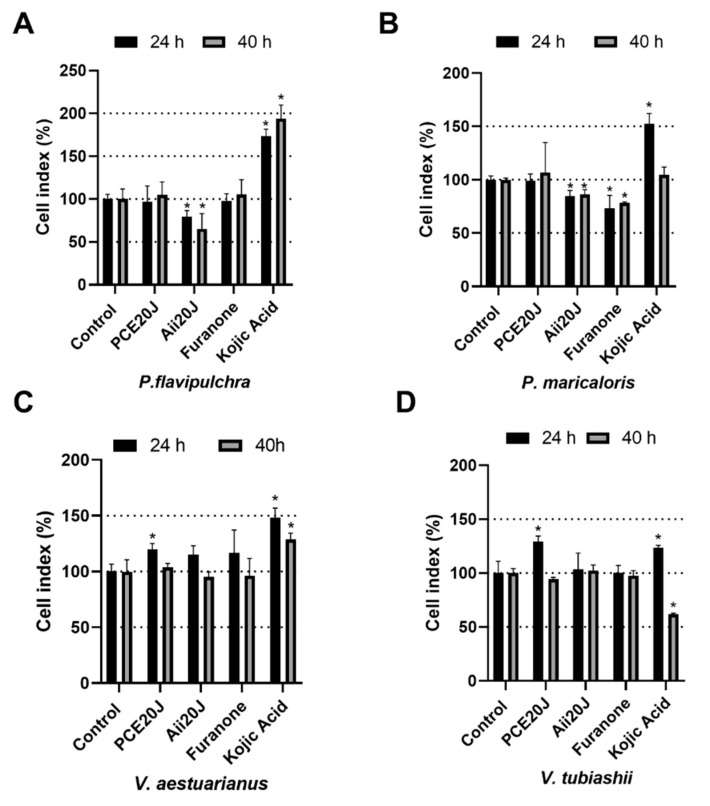
Effect of purified cell extracts, expressed in percentage (%), from *Tenacibaculum* sp. 20J (PCE20J at 100 μg/mL), AHL-lactonase Aii20J (0.57 mM), furanone C30 (0.1 µM), and kojic acid (1 mM) on bacterial biofilm of (**A**) *P. flavipulchra*, (**B**) *P. maricaloris*, (**C**) *V. aestuarianus*, and (**D**) *V. tubiashii* measured by xCELLigence^®^ system (*n* = 3). (*) indicates statistically significant differences (Student’s *t*-test, *p* < 0.05).

In order to check if the xCELLigence^®^ equipment was able to detect changes in the biofilm formation and/or structure caused by QS inhibitors in the selected strains, different inhibitors of AHL-mediated QS were used. The effect of the wide-spectrum QQ enzyme Aii20J [21,22] and the extract from the marine bacterium *Tenacibaculum* sp. 20J, presenting wide-spectrum QQ activity [13], was tested (Figure 3). The QS inhibitors furanone C30 (0.1 µM) [20] and kojic acid (1 mM) [27], used in previous studies, were also tested. 

**Figure 4:**
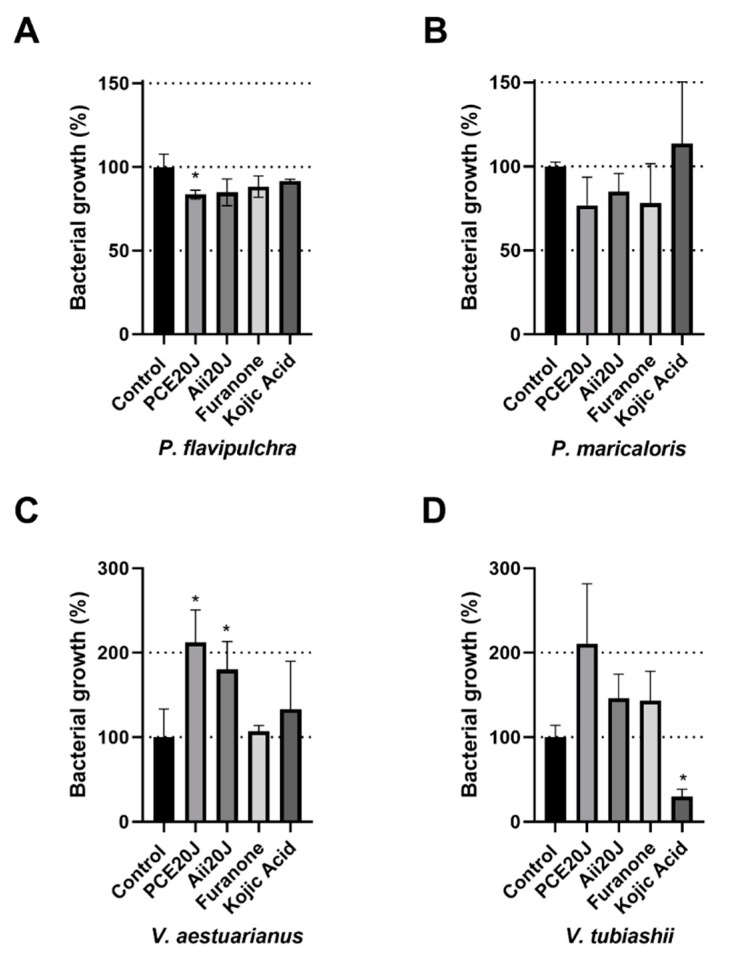
Effect of purified cell extracts from *Tenacibaculum* sp. 20J (PCE20J at 100 μg/mL), AHL-lactonase Aii20J (0.57 mM), furanone C30 (0.1 µM), and kojic acid (1 mM) on planktonic bacterial growth during biofilm formation of (**A**) *P. flavipulchra*, (**B**) *P. maricaloris*, (**C**) *V. aestuarianus*, and (**D**) *V. tubiashii* measured by xCELLigence^®^ system (*n* = 3). (*) indicates statistically significant differences (Student’s *t*-test, *p* < 0.05).

Different responses to QSI compounds were found in the two *Pseudoalteromonas* strains (Figure 3A,B). The AHL-lactonase Aii20J caused a significant reduction of biofilm formed by *P. flavipulchra* (79.47 ± 7.13% and 65.40 ± 17.63% compared with the control biofilm for 24 and 40 h measurements, respectively). Aii20J also inhibited, to a lower extent, biofilm formation in *P. maricaloris* (84.58 ± 5.37% and 86.12 ± 4.63%). The growth inhibition caused by Aii20J in these strains does not seem to be responsible for this biofilm inhibition, since no significant differences were found in planktonic growth between control and treated wells (Figure 4A,B). On the contrary, the furanone only reduced biofilm formation in *P. maricaloris* (73.09 ± 12.23% and 78.27 ± 0.89% for 24 and 40 h measurements, respectively). Despite the high production of AHLs in shaken cultures, the biofilm formed by *V. aestuarianus* was not affected by any of the tested QS inhibitory compounds as measured by xCELLigence^®^. Kojic acid produced significantly increased biofilm formation in most cases, except for *V. tubiashii,* for which significant biofilm inhibition was recorded at 40 h (61.80 ± 1.02%) (Figure 3D), which was probably the result of the planktonic growth inhibition observed (Figure 4D). Since the best specific anti-biofilm results were obtained with the QQ lactonase Aii20J, which reduced the environmental biofilm in two of the four bacterial strains as measured by the xCELLigence^®^, this QQ enzyme was selected for the next experiments.

### 2.5. Effect of the AHL-lactonase Aii20J on Marine Biofilm Formation in the Attachment Model

**Figure 5:**
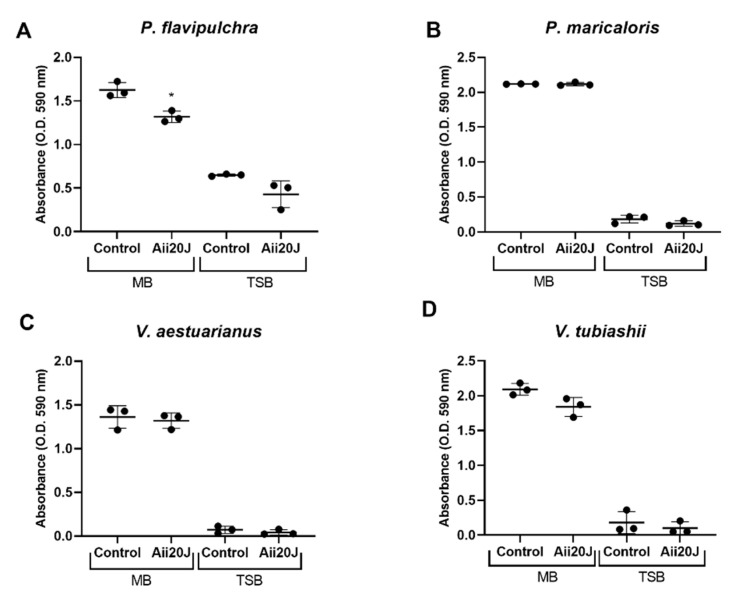
Biofilm production by (**A**) *P. flavipulchra*, (**B**) *P. maricaloris*, (**C**) *V. aestuarianus*, and (**D**) *V. tubiashii* in attachment model, measured by crystal violet staining assay. Effect of AHL-lactonase Aii20J (0.57 mM) on MB and TSB was tested (*n* = 3). (*) indicates statistically significant differences (Student’s *t*-test, *p* < 0.05).

With the aim of comparing the results obtained with the xCELLigence^®^ system, biofilm formation and response to the QQ lactonase Aii20J were assessed with the attachment model for the four selected marine biofilm-forming strains. In this system, biofilm formation was clearly higher in MB than in TSB medium for all species. Confirming the results obtained with the xCELLigence^®^ system, a clear reduction of biofilm formation was observed in *P. flavipulchra* in both MB (81.05 ± 4.01%) and TSB (66.2 ± 27.78%) in the presence of Aii20J (Figure 5A, Supplementary Figure S3). However, no effect was observed in the other bacterial biofilms (Figure 5B–D). 

### 2.6. Effect of the AHL-lactonase Aii20J on Mixed-Species Biofilm Models

**Figure 6:**
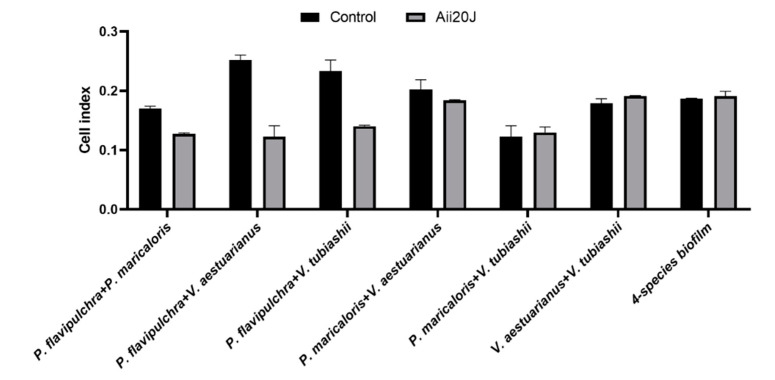
Effect of AHL-lactonase Aii20J (0.57 mM) on two-species and multispecies biofilm formation. Biofilm formation was monitored using xCELLigence^®^ system. Cultures were carried out in TSB. Represented values correspond to cell index after 40 h of incubation (*n* = 2).

The effect of adding the AHL-lactonase Aii20J was assessed on biofilms formed by combinations of the four selected bacteria in pairs using the xCELLigence^®^ monitoring system. The addition of Aii20J (0.57 mM) clearly inhibited biofilms containing *P. flavipulchra* (Figure 6). These data could indicate not only the influence of Aii20J on biofilm formation in the target strain, *P. flavipulchra*, but also an indirect effect of the QQ enzyme Aii20J on the other co-cultured bacteria. However, no effect was observed when the four marine bacteria were cultured together. The co-cultures of various strains were not directly related with higher CI values.

### 2.7. Effect of AHL-lactonase Aii20J on Motility

**Figure 7:**
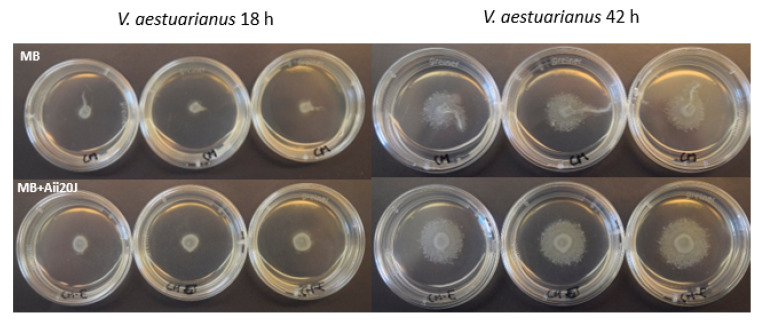
Effect of adding AHL-lactonase Aii20J (0.57 mM) on surface-associated motility in *V. aestuarianus*. Plates were prepared in MB with 0.25% Eiken agar and incubated at 22 °C for 18 and 42 h.

Since surface attachment and biofilm formation are known to be related to surface-associated motility [12], we explored the response of this phenotype to the QQ enzyme Aii20J in order to compare it to the effect observed on biofilm formation. A preliminary experiment was performed to observe the capability of the four selected strains for surface-associated motility in the different culture medium plates (Supplementary Figures S4 and S5). Only *V. aestuarianus* showed a slight surface-associated tentacle-like motility phenotype in MB plates. The addition of the AHL-lactonase (0.57 mM) to the culture medium caused a slightly different motility pattern at 18 and 42 h of incubation, as the tentacle-like type motility observed in the control plates disappeared when the cultures were treated with the QQ enzyme (Figure 7).

## 3. Discussion

Since biofilm formation is the first step in the biofouling process, numerous reports have evaluated the inhibition of bacterial biofilms for the identification of anti-biofouling compounds [25,36,53,54,55]. An understanding of the mechanisms that can be used as targets to interfere with the bacterial adhesion process and biofilm growth and maturation is essential to develop new non-toxic strategies for the control of microfouling. QS has been proposed as an important target for the development of anti-biofilm strategies, but little information is available regarding its role in marine bacterial strains of environmental origin. It is necessary to identify suitable marine biosensor species models and to validate biofilm cultivation and quantification methods that allow the evaluation of QQ strategies for the inhibition of marine biofilms. 

Most of the biofilm quantification methods present several limitations. In this sense, the xCELLigence^®^ system is unable to work at salinity concentrations higher than 3%. Therefore, two *Vibrio* strains (*V. aestuarianus* CECT625 and *V. tubiashii* CECT631) and two *Pseudoalteromonas* strains (*P. flavipulchra and P. maricaloris*) were selected due to their capacity for biofilm formation at different salinity levels, as measured with xCELLigence^®^. Strong differences in the responses of the tested bacterial strains to culture medium and NaCl concentration were observed with this biofilm measurement system. The influence of culture medium on biofilm thickness, surface coverage, and morphology was previously reported in the marine bacterium *Shewanella algae* [56]. The presence of salts can also affect protein adsorption on material surfaces, promoting the establishment of the extracellular polymeric substances (EPS) that form the biofilm matrix [57]. The availability of specific cations is also important. For example, the presence of Ca^2+^ is needed for the maintenance of biofilm integrity in certain marine *Vibrio* species [58] and plays an important role in the synthesis of class II proteins in *Pseudoalteromonas* sp., which are expressed only in biofilm cells [59]. Moreover, culture medium and salinity can also influence the production of QS molecules [12,22,23], therefore their impact on QS-regulated phenotypes should receive more attention. 

Surprisingly, the four marine strains showed the lowest biofilm formation in MB-2 using the xCELLigence^®^ system. On the other hand, in the attachment model, higher biofilm formation was observed in MB compared to TSB. The different characteristics and attachment substrates used in the two biofilm monitoring systems (polystyrene in xCELLigence^®^ and glass in the active attachment system) could also be involved in the observed differences in biofilm formation depending on the culture medium. xCELLigence^®^ uses gold biosensors placed in the bottoms of the wells of special polystyrene plates, therefore biofilm that forms on the walls and the liquid-air interface cannot be measured. On the contrary, the attachment model quantifies all biofilm that forms on the glass coverslip vertically submerged in the culture medium. Furthermore, the substrate can also affect the adhesion process in relation to culture medium composition. Peptone has been reported to increase bacterial adhesion to polystyrene [60], which may explain the higher response obtained with TSB in the xCELLigence^®^ system, since the concentration of peptone in this culture medium is four times higher than in MB and MB-2. Moreover, the use of a specific culture medium could promote a particular biofilm structure and/or biomass accumulation in the well bottom or liquid–air interface, by changes in pH or other physicochemical parameters, indicating that the appropriate selection of culture conditions and substrates is needed before the screening procedure can be established. 

In order to understand the possible effects of QQ and QSI compounds on biofilm formation in these four marine bacteria, the production of AHL-type QS signals was evaluated, revealing that all of them produced AHLs. *V. aestuarianus* showed the richest AHL profile and very high production of OC10-HSL, which could be detected by the long-chain AHL biosensor *C. violaceum* VIR07. However, *V. tubiashii* only produced short-chain C4-HSL when cultured in MB. Numerous studies described *Vibrio* species in which different AHLs regulate multiple functions and, depending on the strain, QS can be involved in biofilm formation but also in biofilm repression and dispersion [61,62]. Moreover, temperature also affected AHL production in different *Vibrio* strains, with a pattern of decreased synthesis with increased temperature [63]. 

HPLC analysis showed that the marine isolates *P. flavipulchra* and *P. maricaloris* produced six and five different AHLs respectively, and four of them were synthesized by both strains (C4, OC6, C8, and OC10-HSL). Other *Pseudoalteromonas* strains isolated from marine environments were reported to produce AHLs [64,65,66,67]. While the *Pseudoalteromonas* genus is considered a major AHL-producing group [38], the relationship of this type of QS molecule with biofilm formation in these species is not clear and more studies are needed. Moreover, QQ activity against long-chain AHLs was found in both strains; in the case of *P. maricaloris,* it changed depending on the culture medium used (Figure 3). The simultaneous production and degradation of AHLs has been reported in several bacteria and has been related to either self-regulation of the QS system or a competitive strategy by removing non-cognate AHLs produced by other bacteria [22]. 

Although it is known that culture conditions can have a high impact on QS-QQ systems, biofilm formation, and/or surface attachment [12,22,23], little attention is usually paid to this important factor when a methodology is established. Although the QQ activity in *Pseudoalteromonas* species is not new and the presence of QQ genes has been reported in previous studies [64,68,69,70], the effect of the culture medium on QQ capability in this genus has not yet been described, to the best of our knowledge, and deserves more attention. Regarding the *Vibrio* strains, *V. tubiashii* also interfered with C12-HSL, but only when cultured in MB. The effect of the environmental conditions on QQ was also described in *Vibrio* strains. For example, in a previous study, interference with AHLs when using LB as culture medium could only be observed when *V. harveyi* R-21446 was grown at 25 and 30 °C [63]. A better understanding of the role of the environmental factors in QS and QQ activity will help to improve the efficiency and specificity of screening methods, therefore more studies are required to confirm the role of environmental factors in AHL-mediated processes.

Previous studies proposed the use of QS inhibitors as potential anti-biofouling agents [25,26,27] and confirmed the control of biofouling [11,15] as well as other types of biofilm [12,13,14,15] using QQ activity strategies. In order to assess the sensitivity of the four biofilm-forming marine bacteria against different agents with the ability to intercept the QS system, compounds such as PCE20J [71], Aii20J [21], furanone C30 [20], and kojic acid [27] were tested in the xCELLigence^®^ system. Although the four bacteria used as biofilm-forming models produce AHLs, the relationship between AHL synthesis and biofilm formation in these marine strains is not clear. Since adding the QQ enzyme Aii20J had no significant effect on the biofilm formed by *V. aestuarianus* and *V. tubiashii* in any of the monitoring systems and culture media used, our results seem to indicate that in these *Vibrio* strains, AHLs do not have a crucial role in the tested conditions. It should be noted that the Aii20J affected the surface-associated tentacle-type motility in the high AHL-producer *V. aestuarianus* observed in MB supplemented with 0.25% Eiken agar. Motility in *Vibrio* species is needed for surface attachment as a previous step to biofilm formation and allows the signal transduction that regulates the development of the biofilm structure [72]. Therefore, QQ may be effective mainly to prevent the initial bacterial attachment stages in *Vibrio* spp., which may explain the lack of activity observed in mature biofilms. 

On the contrary, the AHL-lactonase Aii20J caused significant biofilm inhibition in *P. flavipulchra* and, to a lesser extent, *P. maricaloris*, as measured with the xCELLigence^®^ system. However, the effect of Aii20J on biofilm formed by *P. maricaloris* was not observed in the active attachment model. The different environmental conditions and the characteristics of these biofilm formation systems could be responsible for the different response of *P. maricaloris* to the addition of the AHL-lactonase Aii20J. In this sense, besides the different attachment materials, the active attachment model allows refreshment of the culture medium with additional nutrients, therefore the biofilm cells that form in this system can mature faster, changing their response to the QQ compounds. Despite these differences in biofilm quantification between the xCELLigence^®^ and active attachment systems, the former seems to be slightly more sensitive, with the ability to detect the important biofilm reduction in *P. flavipulchra* caused by the AHL-lactonase Aii20J, and a reduction in *P. maricaloris* biofilm that could not be detected in the active attachment model. Therefore, the results confirm that the xCELLigence^®^ technology can be reliably used to identify novel anti-biofilm compounds also in marine bacteria, although attention should be paid to the culture medium and incubation conditions selected. Still, the use of several strains and/or different culture media is recommended for screening purposes.

In the xCELLigence^®^ experiments, the furanone C30 had a significant effect only on *P. maricaloris* biofilm. Halogenated furanones are the most studied QS inhibitor compounds, and they can block both AI-2 and AHL QS systems by accelerating LuxR turnover [73,74]. Furanones produced by *Delisea pulchra* [20] are able not only to intercept QS signals in biofilms of *Pseudomonas aeruginosa* at 10 µM [75] and biofilms of *C. violaceum* at 100 µM [76], but also to inhibit a mixed assemblage of marine bacteria at 10 mM [77]. The slight effect of the furanone on biofilm formation observed in this study could be explained by the low concentration of furanone used (0.1 µM), since toxicity was reported at higher concentrations [78].

Kojic acid only affected biofilm formation in *V. tubiashii* after 40 h, while the other QQ/QSI inhibitors had no effect on biofilm formation in this strain, but this effect could be a result of the strong growth inhibition observed (Figure 4) instead of more specific action on the QS system. In this sense, despite the fact that kojic acid’s capacity to inhibit biofilm formation has been associated with QS interference [27], our results indicate that it could be caused by its antibacterial properties [79], as previously described regarding other antimicrobial compounds at sub-MIC concentrations [80,81]. 

Single-species in vitro biofilm models are only partially representative of the complexity of marine microbial communities in which less than 1–5% are cultivable bacteria [40], therefore the use of mixed-species biofilms would be desirable. Despite it being generally accepted that QS-related genes are required for the successful establishment of complex marine microbial communities, the knowledge about their role in symbiosis or competition processes in mixed biofilms is still limited. Recently, treatment of 9-day natural biofilms with different QS signals (C6 and C12-HSL, *Pseudomonas* quinolone signal, and cyclic di-GMP) caused a signal-specific change in the community composition through the promotion of specific species with interrelated signal transduction genes [33]. Also, little is known about the effect of the interference of QS systems on the microbial composition of multispecies biofilms. The use of the QQ enzyme SsoPox as a coating additive was recently reported to significantly decrease biocorrosion in steel coupons after 8 weeks of submersion in water, and this effect was associated with a deep change in the microfouling community [28]. In the present work, adding the AHL-lactonase Aii20J (0.57 mM) clearly inhibited the mixed biofilms containing the QQ-sensitive *P. flavipulchra* strain. These data indicate not only the influence on biofilm formation in the target strain, *P. flavipulchra*, but also an indirect effect of the QQ enzyme on the co-cultured bacteria. Although in our case all model species were AHL producers, we cannot disregard a possible effect of the QQ strategies on complex biofilms in which not all bacteria are AHL producers. Some bacteria that do not produce their own AHL can respond to the addition of AHLs or AHL analogues because of the presence of signal receptors, known as LuxR orphans, that are widespread in marine bacteria [23]. In this sense, the AHL-lactonase Aii20J has been reported to be active against AHL producers [12,22,23], but also against bacterial strains that are not [21]. Moreover, previous studies indicated that the external addition of QQ enzymes that degrade AHLs into complex biofilm could affect both Gram-negative and Gram-positive bacteria [14,28,29,30]. 

Although little attention is usually paid to the culture conditions, this study demonstrates the importance of this crucial factor in QS-QQ systems, biofilm formation, and/or surface attachment. The use of xCELLigence^®^ is proposed as a time-saving method to quantify biofilm formation and search for eco-friendly anti-microfouling compounds based on QS and QSI strategies. However, the use of only low-salinity culture medium is a limitation that should be taken into account and could be overcome by combining methodologies and strains. Additionally, the results of this study reveal important differences in the response of biofilm formation in different AHL producers, suggesting that several strains should be used simultaneously for screening purposes. Further studies are required to ascertain the effect of QS and QSI on natural marine biofilms and their impact on bacterial ecology.

## 4. Materials and Methods 

### 4.1. Bacterial Strains and Growth Conditions

*Cobetia marina* CECT4278, *Shewanella putrefaciens* CECT5346, *Vibrio aestuarianus* CECT625, and *Vibrio tubiashii* CECT631 were obtained from the Spanish Type Culture Collection (CECT). The isolates *P. flavipulchra* and *P. maricaloris* were kindly provided by Prof. Juan Barja at the Universidade de Santiago de Compostela, and *Vibrio anguillarum* 90-11-287 was provided by Prof. Lone Gram at the Technical University of Denmark. All marine bacteria used in this study were routinely cultured at 22 °C on Marine Agar/Broth (MA/MB) at pH 7.0 (Laboratorios Conda S.A., Madrid, Spain) and/or Tryptone Soy Agar/Broth (TSA/TSB) (Scharlau S.L., Barcelona, Spain). TSB (NaCl concentration 0.5%) was supplemented with NaCl to achieve a concentration of 2% (TSB-2) (total salt concentration 2.25%). Modified Marine Broth (MB-2) was prepared by diluting standard MB with distilled water to adjust the total salt concentration to 2.25%, and peptone and yeast extract were added to maintain the original nutrient concentration. The QQ *Tenacibaculum* sp. strain 20J CECT7426, isolated in our lab from a marine sample [82], was routinely cultured at 22 °C on Marine Agar/Broth at pH 7.0. 

The AHL biosensor strains *Chromobacterium violaceum* CV026 [83] and VIR07 [84] were used for AHL production and quorum quenching activity bioassays in solid plates, as explained below. These strains were routinely cultured on Luria-Bertani (LB) medium supplemented with kanamycin (50 μg/mL) at 30 °C. 

### 4.2. Bioassay for Detection of AHL Production in Marine Cultures

The marine bacterial strains were screened for their capability to activate the AHL biosensors *C. violaceum* CV026 [83] and VIR07 [84]. The strains were cultured in Eppendorf tubes in 500 μL of MB, MB-2, TSB, or TSB-2 for 24 h. The cultures were centrifuged (Galaxy 7D microcentrifuge, VWR, Radnor, PA, USA), and the supernatants were transferred to TSA plates covered with an overnight culture of *C. violaceum* CV026 or VIR07 diluted in soft (0.8%) agar (1:5). The plates were incubated for 24 h at 30 °C, and the production of purple coloration around the wells was checked. PBS at pH 6.5 with the corresponding AHL (10 μM) was used as control. 

### 4.3. Extraction and Identification of AHLs by HPLC-MS

AHL production was also assessed by HPLC-MS (6495C Triple Quadrupole LC/MS, Agilent, Santa Clara, CA, USA) analysis from supernatants of *V. aestuarianus* CECT625, *V. tubiashii* CECT631, *P. flavipulchra*, and *P. maricaloris* cultured in MB at 22 °C and 200 rpm for 24 h. Culture samples of 40 mL were taken and centrifuged (9000× *g*, 10 min) to obtain the supernatants. Remaining AHLs in supernatants were extracted twice with 40 mL of dichloromethane, and this solvent was evaporated to dryness in a rotary evaporator system at 40 °C. AHLs produced by the bacteria were reconstituted in 1 mL of acetonitrile and quantified by HPLC-MS [14,85]. Pure AHLs, both C3 hydroxy- and oxo-substituted and un-substituted, were obtained from Sigma-Aldrich and the University of Nottingham and used as external standard for quantification.

### 4.4. Quorum Quenching Activity Assay

The QQ activity of the 4 selected marine strains was tested using solid plate assays [71] carried out with the AHL biosensors *C. violaceum* CV026 [83] and VIR07 [84]. The marine strains were cultured in Eppendorf tubes in 500 μL of MB, MB-2, TSB, or TSB-2 for 24 h. The cultures were centrifuged, the supernatants were removed, and the pellets were washed with 500 μL of phosphate-buffered saline (PBS) at pH 6.5 and resuspended in another 500 μL of the same buffer in order to avoid spontaneous lactonolysis of the exogenous AHLs by high pH values [86]. These cell suspensions were used for a live-cell AHL degradation assay by adding C6-HSL (10 µM) or C12-HSL (10 µM) and incubating for 24 h at 22 °C. After the incubation period, the live-cell suspensions were centrifuged, and the presence of AHLs in the supernatants was tested as explained above. 

### 4.5. Quorum Sensing Inhibitors

Purified cell extract (PCE) from *Tenacibaculum* sp. 20J, which has broad QQ activity against AHL and AI-2 [13,71], was obtained from a 50 mL MB culture that was centrifuged for 5 min at 2000× *g* in order to separate the biomass from the culture medium. The pellet was washed with PBS at pH 6.5, resuspended in 1.5 mL of the same buffer, sonicated for 5 min in ice, and centrifuged at 16,000× *g* for 30 min at 4 °C. The PCE was filtered through a 0.22 μm filter and stored at 4 °C [87] until it was used at a final concentration of 100 μg/mL.

The QQ enzyme Aii20J was produced and purified as previously described [21]. Briefly, the *E. coli* BL21(DE3) plysS strain expressing recombinant protein was inoculated into fresh LB medium with kanamycin (25 µg/mL) at 37 °C. Protein expression was induced when the culture reached an optical density (OD) of 0.6 by the addition of 0.1 M isopropyl-d-thiogalactopyranoside (IPTG), followed by further incubation for 5 h. Then, the culture was centrifuged, and the pellet was resuspended with 20 mL of PBS buffer, lysed by sonication on ice, and centrifuged again. Aii20J was purified using the His GraviTrap affinity column (GE Healthcare) protein purification kit and dialyzed in a d-Tubes Dialyzer Mega MWCM 6–8 KDA (GE Healthcare).

The QS inhibitors furanone [20] and kojic acid [27], purchased from Sigma, were dissolved in PBS at pH 6.5 and checked at final concentrations of 0.1 and 1 mM, respectively. 

### 4.6. Biofilm Quantification 

In order to determine the ability of the xCELLigence^®^ System RTCA DP (ACEA Biosciences Inc., San Diego, CA, USA) to quantify the biofilm formed by the marine bacteria, 24 h cultures of the bacteria *V. anguillarum* 90-11-287, *V. aestuarianus* CECT625, *V. tubiashii* CECT631, *C. marina* CECT4278, *S. putrefaciens* CECT5346, *P. flavipulchra*, and *P. maricaloris* in MB were adjusted to 0.05 OD_600_ in the appropriate culture medium (TSB, TSB-2, or MB-2) and mixed well, and then 180 μL was added into E-plates 16 (ACEA Biosciences Inc., San Diego, CA, USA). In the biofilm inhibition experiments, a volume of 20 μL of the adequate dilution of compounds PCE20J, Aii20J, furanone, and kojic acid was added to the cultures to achieve 100 µg/mL, 0.57 mM, 0.1 μM, and 1 mM, respectively. The mixture was incubated at 22 °C and cell index data were recorded for 40 h. As controls, culture media inoculated with biofilm-forming bacteria and 20 μL of PBS were used. Experiments were performed in triplicate.

Biofilms were also grown in the attachment model [48], a modification of the Amsterdam Active Attachment (AAA) model [47,48], assembled with glass coverslips (18 × 18 mm). Quickly, coverslips were vertically submerged in 3 mL of culture medium inoculated with 24 h shaken cultures of the selected marine strains at an optical density of 0.05 (OD_600_ nm) in 12-well culture plates. Biofilms were grown at 22 °C for 24 h, with medium refreshment (12 h) as described previously [48]. The QQ enzyme Aii20J at a final concentration of 0.57 mM was added to the cultures. Wells with only culture medium were incubated in the same conditions as negative growth controls. Three replicates were performed for each strain or treatment. For biofilm biomass quantification, the coverslips were removed after incubation, deposited in clean 12-well culture plates, and allowed to dry. Once dried, the wells were filled with crystal violet solution (0.04%, Gram-Hucker, Panreac), and after 20 min, the excess dye was removed, and coverslips were washed several times with distilled water. Bound crystal violet was released by adding 33% acetic acid. The absorbance (Helios Omega UV-Vis Spectrophotometer, Thermo Scientific, Waltham, MA, USA) was measured at 590 nm [88]. 

### 4.7. Motility Assays

Surface-associated motility assays were performed as described by Mayer et al. [22]. Petri dishes were prepared with MB, MB-2, TSB, and TSB-2 supplemented with 0.25% Eiken agar (Eiken Chemical Co. Ltd., Tokyo, Japan). The AHL-lactonase Aii20J was mixed with the culture media at a concentration of 0.57 mM. One microliter from shaken overnight cultures at 0.3 optical density (OD_600_ nm) was inoculated in the center of the plates. Plates were incubated at 22 °C in the dark, and surface-associated motility was inspected after 18 and 42 h. Three plates were prepared for each condition.

### 4.8. Statistical Methods

Student’s *t*-test (*p* < 0.05) was applied for all statistical analyses using GraphPad Prism (Version 8.3.0, GraphPad Software, San Diego, CA, USA).

## Figures and Tables

**Table 1:** HPLC-MS quantification (ng/mL) of acyl-homoserine-lactones (AHLs) produced by biofilm-forming bacteria cultured in MB using HPLC-MS. (-): not detected in the sample.

	*P. flavipulchra*	*P. maricaloris*	*V. aestuarianus* CECT625	*V. tubiashii* CECT631
C4-HSL	374	347	626	503
OC6-HSL	2.31	0.49	0.8	-
C7-HSL	1.06	-	-	-
C8-HSL	0.74	0.66	39.2	-
OC8-HSL	-	0.46	15	-
C10-HSL	0.68	-	34.3	-
OC10-HSL	5.79	1.13	2958	-
HC10-HSL	-	-	26.47	-
C12-HSL	-	-	2.97	-
OC12-HSL	-	-	41.66	-
HC12-HSL	-	-	3.32	-
C16-HSL	-	-	2.2	-

## Supplementary Materials

The following are available online at https://www.mdpi.com/1660-3397/19/2/74/s1, Figure S1. Biofilm formation of the marine bacterial biosensor measured by xCELLigence® system using the culture media TSB. Figure S2. Capability of the four selected strains (A) *P. flavipulchra*, (B) *P. maricaloris*, (C) *V. aestuarianus* and (D) *V. tubiashii* to activate the quorum sensing-regulated violacein production by *C. violaceum* VIR07 using different culture media. Figure S3. Effect of the addition of the AHL-lactonase Aii20J (0.57 mM) on biofilm formation by *P. flavipulchra* using the attachment model. Figure S4. Effect of the culture media on surfacce-associated motility in *P. flavipulchra* and *P. maricaloris.* Figure S5. Effect of the culture media on surfacce-associated motility in *V. aestuarianus* and *V. tubiashii.*
