# Supplementary material for: Use of Quorum Sensing Inhibition Strategies to Control Microfouling
Source: Mar Drugs. 2021 Jan 30;19(2):74. doi: 10.3390/md19020074 (PMC7912365; doi:10.3390/md19020074)
Supplement: Supplementary file 1 [file marinedrugs-19-00074-s001.pdf]

marine drugs

Supplementary Materials

Use of quorum sensing inhibition strategies to control microfouling

Andrea Muras, Ana Parga, Celia Mayer and Ana Otero*

Departamento de Microbioloxía e Parasitoloxía, Facultade de Bioloxía-CIBUS, Universidade de Santiago de Compostela, 15782 Santiago de Compostela, Spain

* Correspondence: anamaria.otero@usc.es; Tel.: +34-881-816-913

Received: date; Accepted: date; Published: date

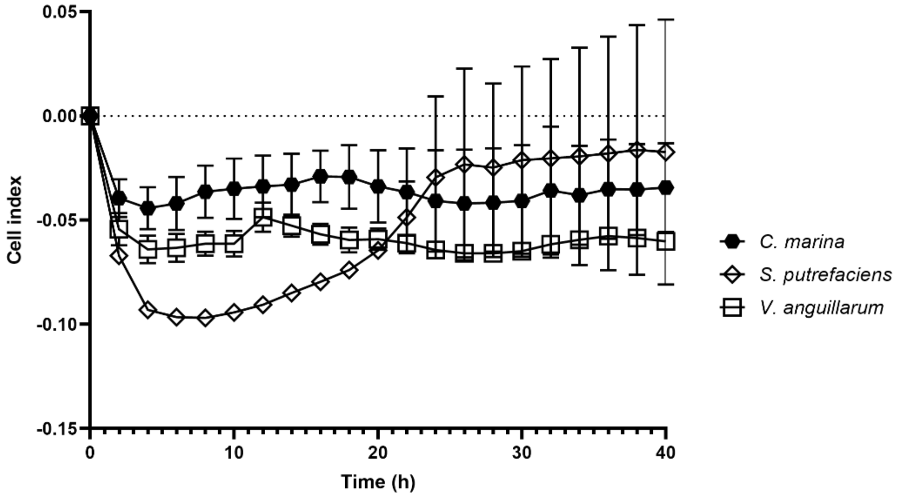

Figure S1. Biofilm formation of the marine bacterial biosensor measured by xCELLigence® system using the culture media TSB.

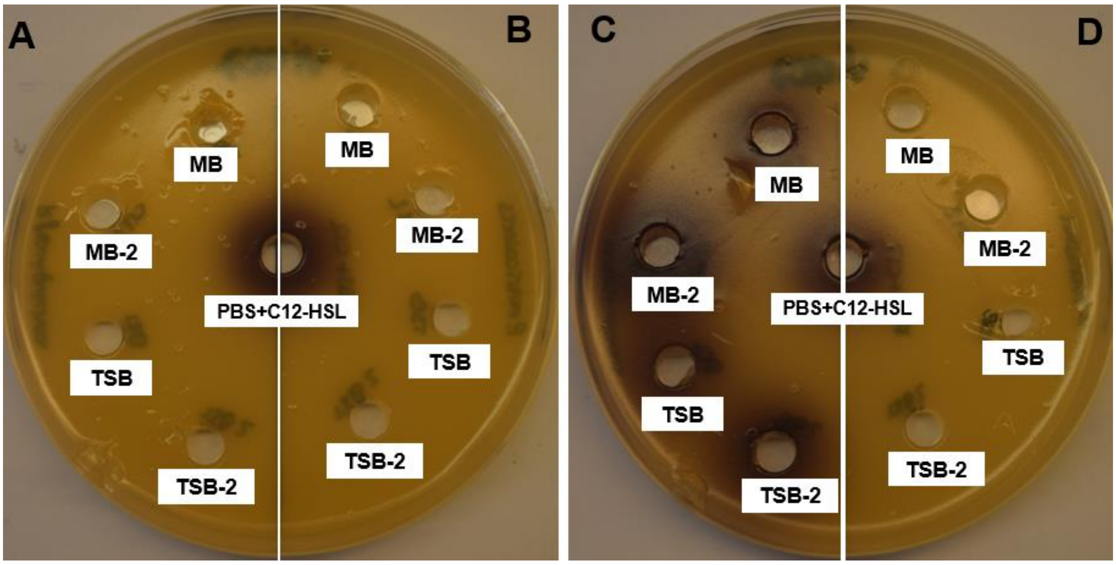

Figure S2. Capability of the four selected strains **(A)** *P. flavipulchra*, **(B)** *P. maricaloris*, **(C)** *V. aestuarianus* and **(D)** *V. tubiashii*to activate the quorum sensing-regulated violacein production by *C. violaceum* VIR07 using different culture media.

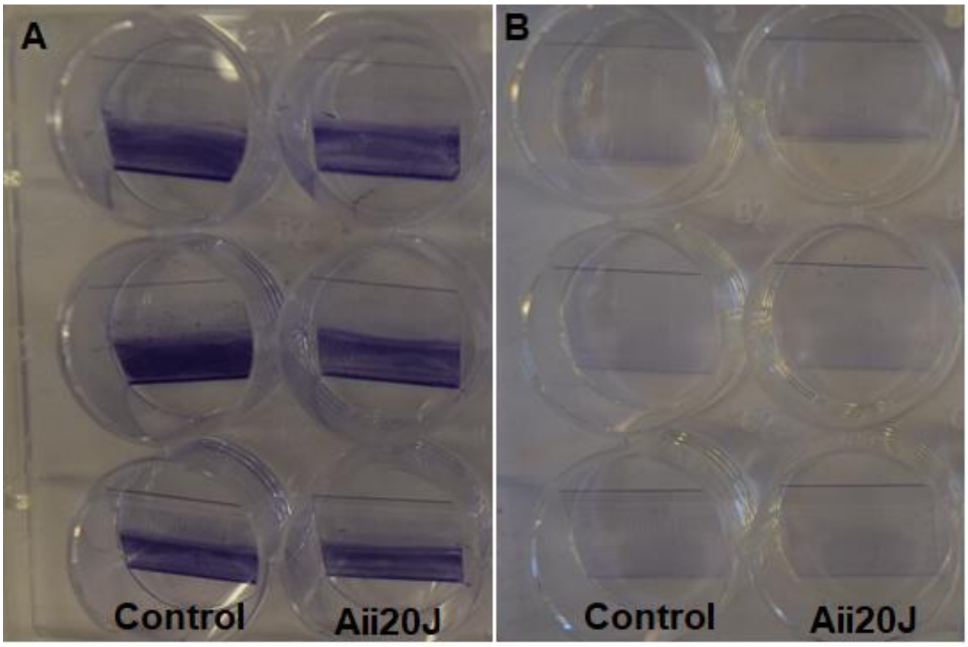

Figure S3. Effect of the addition of the AHL-lactonase Aii20J (0.57 mM) on biofilm formation by *P. flavipulchra* using the attachment model. The biofilms were cultured in **(A)** MB and **(B)** TSB and incubated 22ºC for 24h.

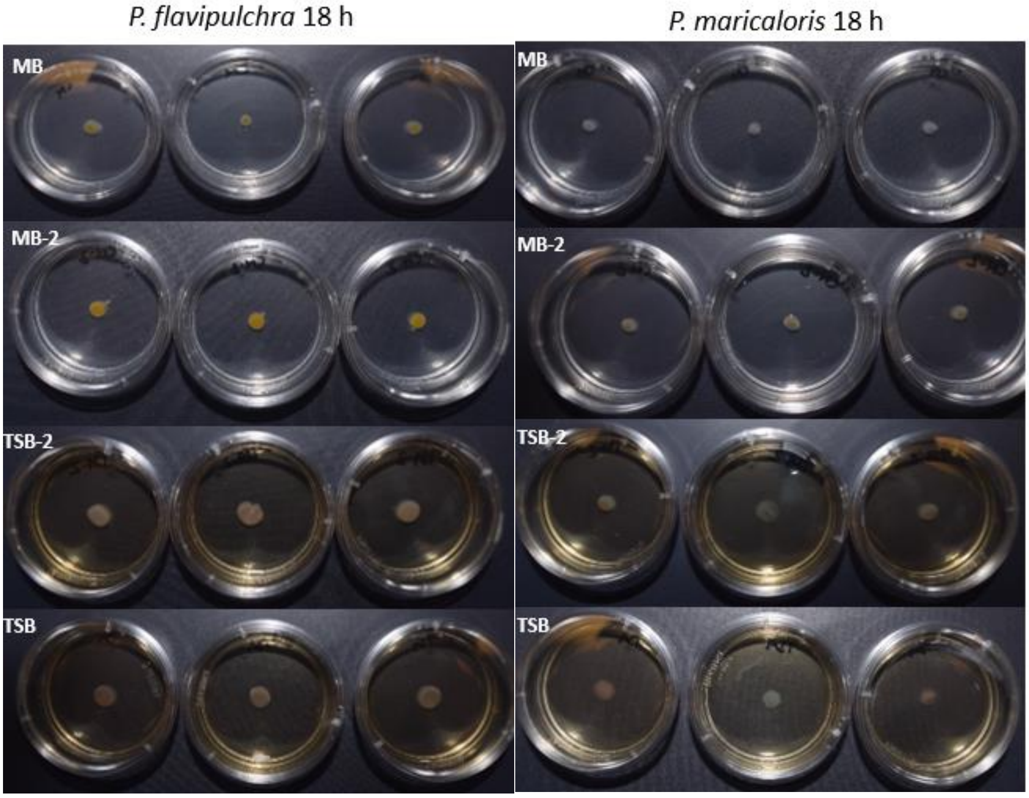

Figure S4. Effect of the culture media on surfacce-associated motility in *P. flavipulchra* and *P. maricaloris*. Plates were prepared in MB, MB-2, TSB-2 and TSB with 0.25% Eiken agar and were incubated at 22°C for 18h.

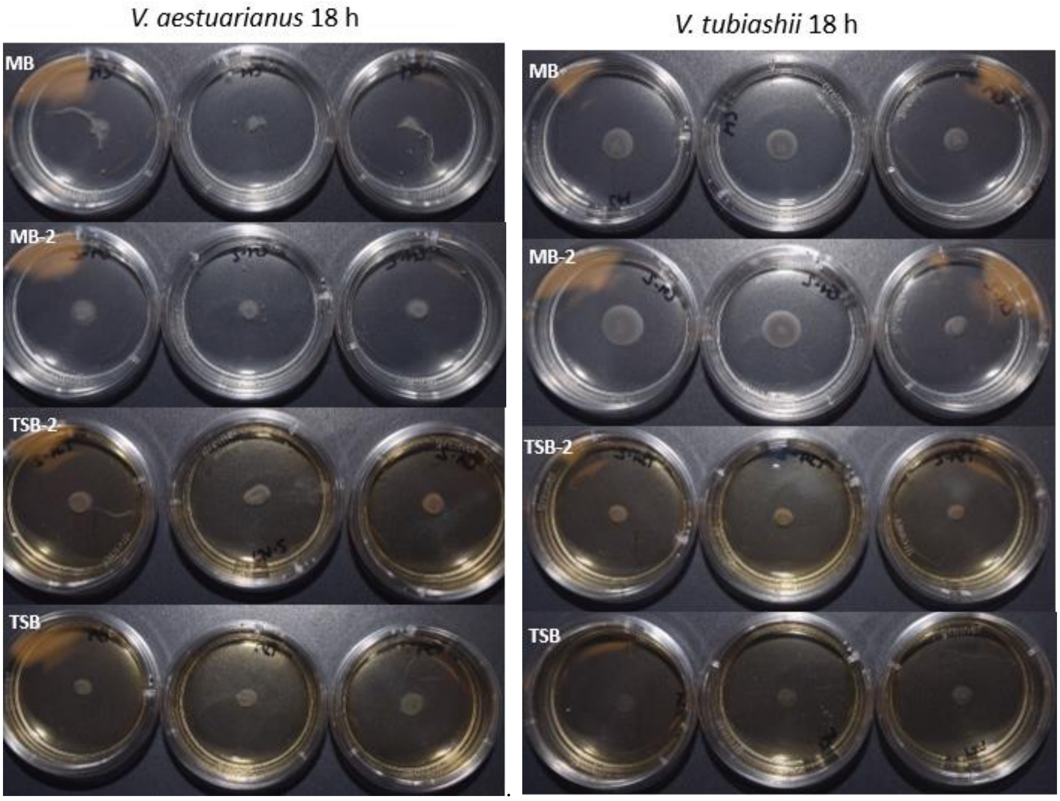

Figure S5. Effect of the culture media on surfacce-associated motility in *V. aestuarianus* and *V. tubiashii.* Plates were prepared in MB, MB-2, TSB-2 and TSB with 0.25% Eiken agar and were incubated at 22°C for 18h.

Publisher's Note: MDPI stays neutral with regard to jurisdictional claims in published maps and institutional affiliations.

© 2021 by the authors. Submitted for possible open access publication under the terms and conditions of the Creative Commons Attribution (CC BY) license (http://creativecommons.org/licenses/by/4.0/).
